# The Use of Thermol in Influenza and Distemper in the Dog and Cat

**Published:** 1901-09

**Authors:** H. B. Felton

**Affiliations:** Philadelphia, Pa.


### THE USE OF THERMOL IN INFLUENZA AND DISTEMPER IN THE DOG AND CAT.

By H. B. Felton, B.S., V.M.D.,
PHILADELPHIA, PA.

My attention was first directed to thermol by several of my friends in the medical profession who had used it with rather remarkable results in typhoid fever, la grippe, and pneumonia. Notably, Dr. George B. Miller, of Philadelphia, associate editor of the *Medico-Legal Journal*, of New York, whose article on the use of thermol in typhoid fever, appearing in the *Philadelphia Medical Journal* of September 30, 1899, first called the attention of the medical profession to this remedy.

Thermol belongs to the family of anilides, and is a true definite and permanent chemical compound or alkaloid, having the formula $C_{14}H_{15}NO_3$. It is a white, crystalline, odorless and tasteless powder, very slightly soluble in cold water, more so in boiling water, and very soluble in alcohol. It is the best dissipator of the aromatic series of synthetic drugs, because all of the phenyl or aniline toxic properties have been entirely eliminated from it in the various stages of manufacture. Thus thermol is not a heart depressant, being absolutely safe to use, even in the presence of cardiac disease, when in need of an antipyretic.

Thermol is a true thermotoxic, and acts by restoring the normal heat-regulating powers of the nervous system. It prevents the formation of heat by stopping increased tissue combustion through its sedative and controlling influence over the heat-controlling nerve centre. It increases arterial pressure by stimulating the heart and vasomotor system.

Thermol also possesses a marked analgesic action, and its nerve-calming and soothing effects are very marked in typhoid fever and la grippe.

In addition to these actions thermol seems to have the property of either inhibiting germ cultures in the blood or of antagonizing the leucomaines that are formed. In other words, its action is that of an antiseptic in the blood. By reason of this action we are able to abort many cases of disease where it is administered at the beginning.

Having heard such good results from thermol in the diseases above mentioned, I argued that it ought to be a good thing to use in influenza and distemper in dogs and cats, and I am happy to state that an experience of about nine months with this remedy justifies me in saying that it has stood the test and possesses all the good qualities that have been claimed for it.

In conjunction with Dr. Hartman, at his infirmary, I have been able to study the action of thermol in about fifty cases of influenza in dogs, about thirty cases in cats, and ten cases of distemper in dogs.

Since 1892 influenza has been more or less epidemic among cats and dogs in Philadelphia. Whether the germ that produces la grippe in man is responsible for this disease we believe has never been determined, but it is certain that the same atmospheric conditions seem to produce it, and it runs a course similar in many respects to influenza in the human subject, and with similar complications.

In the fall of 1892 I noticed the first outbreak in the infirmary which I was conducting at that time. After a very hot summer, during which I boarded quite a number of dogs and cats, the weather suddenly grew quite cool overnight, and continued so for several days. On the second day of the change all the dogs and cats were sneezing and coughing and exhibiting well-marked symptoms of influenza, which ran a course of from two to three weeks, and left a number of patients in very bad shape. This is but a sample of a number of outbreaks that have occurred, and notwithstanding thorough cleansing, whitewashing, and fumigating with sulphur, burning tar, etc., we found that the germs retained their vitality for several weeks, and new animals coming in were liable to contract the disease.

The most stubborn complication that we have had to deal with in dogs has been the laryngeal, indicated by a very severe paroxysmal cough, coming on about the second day and persisting, in many cases, in spite of all the remedies we could employ, or that our medical friends could suggest, for two or three weeks, until the patient became thoroughly worn out, and in some cases died from exhaustion.

We have noticed that the abortive action of thermol is very marked in influenza in the dog. If administered in two and one-half grain doses every three hours at the onset, when there is a watery discharge from the eyes and nose, and sneezing, the symptoms will quickly subside, and there will be complete recovery in from two to three days, while the result of former treatment was that the disease would run a course, more or less severe, of from two to three weeks.

In cases where the paroxysmal cough has developed, which we believe is largely of nervous origin, the action of the thermol is most happy. Given in five-grain doses every three hours in severe cases, and in two and one-half in milder cases the cough either ceases in from twenty-four to forty-eight hours or becomes soft and moist in character, and ceases to distress the patient.

One case may be cited. A cocker spaniel dog, about two years old, was brought to the infirmary suffering from influenza complicated with double pneumonia. A very grave prognosis was given, and no hope of recovery was entertained, as there was high fever, great weakness and depression, and complete loss of appetite. At the request of the owner, however, treatment was undertaken, which consisted of five grains of thermol every three hours for the first three days and milk-punches given in small quantities and frequently repeated.

On the second morning the temperature was down to 101°, and the patient perceptibly brighter. By the third day there was a decided improvement, the lungs beginning to clear up, and the fever kept under control by the continued use of thermol, which, after this time, was given in two and one-half grains every four hours for about a week. The animal made a good recovery, and was discharged at the end of the second week.

This was probably the most severe of the fifty cases of influenza we treated, all of which recovered.

Our experience in the treatment of influenza in cats was unsatisfactory prior to the use of thermol.

During the Christmas season of 1897 and 1899 one of our large department stores had for sale a large number of Angora cats. On both occasions there was a severe outbreak of influenza among these animals, which we were called to treat, and a mortality of fully 50 per cent. There was sneezing, discharge from the eyes and nose, and high fever, which, in many cases, rapidly produced a deep stupor and death in from twenty-four to forty-eight hours. In other cases there were complications of laryngitis, rapidly developing into bronchitis and pneumonia, which was equally fatal.

Probably these two outbreaks were more severe than any we have had to treat subsequently, but we are confident that had we employed thermol we would have reduced the percentage of deaths very materially.

The stubborn symptoms in cats are the persistent sneezing, corresponding to the paroxysmal cough in dogs, and the discharge from the eyes and nose.

We find that two grains of thermol given every three hours will stop the sneezing in from two to three days, when given at the outset, and most cases will recover very nicely in from a week to ten days, with a gradual subsidence of all the symptoms.

In distemper in dogs our experience with thermol has been very satisfactory. Of ten cases treated all recovered.

Thermol, given in the commencing stages, materially checks the course of the disease and modifies the symptoms.

When the disease has become firmly established thermol controls the fever, relieves the consequent depression, and checks the undue tissue waste. The tongue becomes moist and cool, and in most cases the appetite returns with the reduction of the fever.

The antiseptic action of thermol in the bowels is quite marked, causing the offensive odor of the feces to disappear very quickly. In some cases it controls the diarrhœa very nicely without the use of other remedies, while in a few cases we have noticed that it increases peristalsis, not, however, increasing the fluidity of the stools. In none of the cases treated were there any marked nervous complications, although in two or three instances these might have been expected from the temperament and breeding of the dog. Whether thermol will prevent these nervous complications in all cases, or what its action will be upon these complications, yet remains to be determined.

While we do not claim that thermol is a specific, yet we do claim that we have found it to be of great value in the treatment of the diseases enumerated, and we treat these diseases with greater confidence and better results than formerly, and if our results shall be confirmed by the experience of others, we shall feel that we have introduced a drug that will be of great value to our profession.

